# Transcriptomic analysis of genes in soybean in response to *Peronospora manshurica* infection

**DOI:** 10.1186/s12864-018-4741-7

**Published:** 2018-05-18

**Authors:** Hang Dong, Shuangfeng Shi, Chong Zhang, Sihui Zhu, Mei Li, Jie Tan, Yue Yu, Liping Lin, Shirong Jia, Xujing Wang, Yuanhua Wu, Yuhui Liu

**Affiliations:** 1 Biotechnology Research Institute, Chinese Academy of Agricultural Sciences, Beijing, China; 2 College of Plant Protection, Shenyang Agricultural University, Shenyang, China; 3 School of Biotechnology, East China University of Science and Technology, Shanghai, China

**Keywords:** Soybean downy mildew, RNA-seq analysis, Differentially expressed genes, Transcription factors, Disease resistance, Soybean

## Abstract

**Background:**

Soybean downy mildew (SDM), caused by *Peronospora manshurica (Pm),* is a major fungal disease in soybean. To date, little is known regarding the defense mechanism at molecular level and how soybean plants response to *Pm* infection. In this study, differential gene expression in SDM-resistant (HR) and SDM-susceptible (HS) genotype was analyzed by RNA-seq to identify differentially expressed genes (DEGs) following *Pm* infection.

**Results:**

Of a total of 55,017 genes mapped to the soybean reference genome sequences, 2581 DEGs were identified. Clustering analysis of DEGs revealed that these genes could be grouped into 8 clusters with distinct expression patterns. Functional annotation based on gene ontology (GO) and KEGG analysis indicated they involved in diverse metabolism pathways. Of particular interest were the detected DEGs participating in SA/ROS and JA signalling transduction and plant/pathogen interaction.

**Conclusion:**

Totally, 52 DEGs with *P* value < 0.001 and log_2_ fold change > 2 or < − 2 upon fungal inoculation were identified, suggesting they were SDM defense responsive genes. These findings have paved way in further functional characterization of candidate genes and subsequently can be used in breeding of elite soybean varieties with better SDM-resistance.

**Electronic supplementary material:**

The online version of this article (10.1186/s12864-018-4741-7) contains supplementary material, which is available to authorized users.

## Background

Soybean (*Glycine max* L.) is a major crop worldwide that provides abundant amount of protein and oil for human and animal consumption. However, soybean was severely affected by an obnoxious fungal disease, downy mildew (SDM), caused by *Peronospora manshurica* (*Pm*). The yield loss could be as high as 6–15% on average in the years when it was epidemic [1–4]. The proliferation of this fungus was favored by high humidity and relatively low temperature at 20–22 °C. Initially infected leaves had pale green to light yellow spots on the upper surface but later on these spots were creamy white to pinkish coupled with growth of fruiting bodies on the back side of leaves leading to chlorophyll degradation and premature defoliation. In addition, the fungi obtain nutrients exclusively from living plant cells and cannot be cultured apart from their host [5, 6].

Although some fungicides can be applied to reduce SDM infection, the most effective way to control this disease and reduce yield loss is to develop and use resistant variety in soybean production. In our previous study [7], 15 soybean genotypes were either in vitro disease-indexed via leaf/*Pm* inoculation or by field observation in two consecutive years. By which the genotypes were classified into five categories: HR (highly resistant), R (resistant), MR (moderate resistant), S (susceptible) and HS (highly susceptible). We had cloned a *GmSAGT*1 gene coding for a bi-functional enzyme of serine/alanine glyoxylate aminotransferase from a soybean genotype highly resistant to SDM. Fifteen soybean genotypes were used for qRT-PCR analysis, which showed the average expression level of *GmSAGT*1 in HR group was 13 times of that in HS group. Northern blot analysis indicated that the expression level of *GmSAGT*1 in HS, S, MR and R group was 5, 22, 39 and 65% of that in HR, respectively. Transgenic tobacco expressing *GmSAGT*1 increased resistance to a fungal pathogen *Rhizoctonia solani,* demonstrating that higher expression of *GmSAGT*1 gene was closely correlated with increased SDM resistance.

However, the genomic mechanism associated with SDM resistance is currently not known that needs to be further elucidated. Understanding the genetic regulation of plant defense system will help to identify specific genes for either gene-based marker selection or genetic engineering to develop soybeans with better disease resistance. With the advent of next-generation sequencing technology, Illumina sequencing approach has been used for understanding the complexity of gene expression and regulation networks in plants responding to different pathogen attack, including bacterial leaf pustule (*Xanthomonas axonopodis* pv. *glycines*) [8, 9], Phytophthora root and stem rot (*Phytophthora sojae)* [10], Asian soybean rust (*Phakopsora pachyrhizi*) [11] in soybean, and soybean cyst nematode (SCN; *Heterodera glycines*) in common bean [12]. By which the responsive genes and metabolism pathways associated with disease resistance have been identified. Unfortunately, no such a study on soybean/*Pm* interaction has yet been reported so far.

To gain transcriptional profiling data after *Pm* infection, a highly SDM-resistant genotype (HR) Jilinxiaoli1 (JL1) and a highly susceptible (HS) genotype Kefeng1 (KF1) were used for comparison in this study. The differentially expressed genes of HR and HS at 72 h after inoculation (hai) was compared with that obtained from non-inoculated leaves. Totally, 52 DEGs with *P* value < 0.001 and log_2_ fold change > 2 or < − 2 upon fungal inoculation were identified. Of particular interest was the detected DEGs might involve in SA (salicylic acid), ROS (reactive oxygen species) and jasmonic acid (JA) signaling, implicating their roles in response to *Pm* invasion. A high level of accumulation of defense responsive gene products such as pathogenesis-related (PR) proteins and NBS-LRR (nucleotide binding site-leucine rich repeat) might also contribute to SDM-resistance in soybean. In addition, some transcription factors (TFs) of bHLHs, MYBs and WRKYs were up-regulated after inoculation. These findings provided an insight for further functional characterization of candidate genes resistant to SDM that would help in improving soybean breeding program. To the best of our knowledge, this is the first report describing differential expression of genes in response to soybean/*Pm* interaction.

## Results

### SDM symptoms

**Fig. 1:**
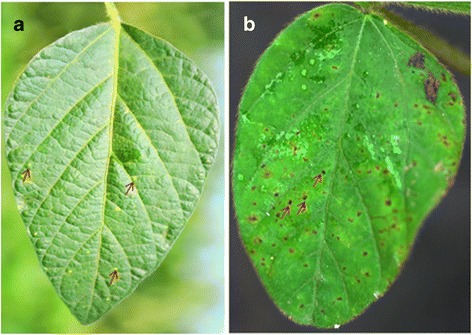
SDM-symptoms on leaves of HR/JL1 and HS/KF1. **a** & **b**: showing SDM-symptoms on leaves of JL1 and KF1 72 h after inoculation (hai) with fungal pathogen *P. manshurica*

Symptoms on leaves of JL1 (HR) and KF1 (HS) were investigated 72 hai. There were a large number of brown lesions surrounded by yellow haloes on the surface of leaves of HS KF1. In contrast, only a few number of small yellow spots on the leaves of HR JL1 formed (Fig. 1). The behavior of HR and HS genotype after inoculation was as expected, indicating their leaf samples could be used for further transcriptomic analysis.

### RNA-seq analysis

Changes in transcript levels between inoculated and non-inoculated (JL1i vs. JL1ni and KF1i vs. KF1ni) were analyzed by RNA-seq. A total of 128.22 million raw reads were generated by 75 bp single end sequencing from the four cDNA libraries, constituting 4.51 Gb of cDNA sequences per library on average (Additional file 1: Table S1). By trimming adaptor sequences and elimination of low quality reads as well as reads containing unknown nucleotides larger than 10%, a total of 120.07 million clean reads were generated. Of which, 97.00 and 95.75 million clean reads were mapped (80.78%) and uniquely mapped (79.47%), respectively, to the soybean genome reference sequence (*Glycine max* Wm82.a2.v1) by using TopHat software (Additional file 2: Table S2). Of 65,781 predicted genes in the soybean genome, the expression level of 55,017 mapped genes were quantified based on RPKM values (reads per kilo bases per million reads) converted from mapped read counts of DEGs.

### Transcriptome analysis in response to *Pm*-inoculation

Using the random sampling model in the DEGseq program, the mapped read counts of each gene with a *P*-value < 0.001 and log_2_ fold change > 2.0 or < − 2.0 were selected, by which a total of 2581 DEGs were obtained.

**Fig. 2:**
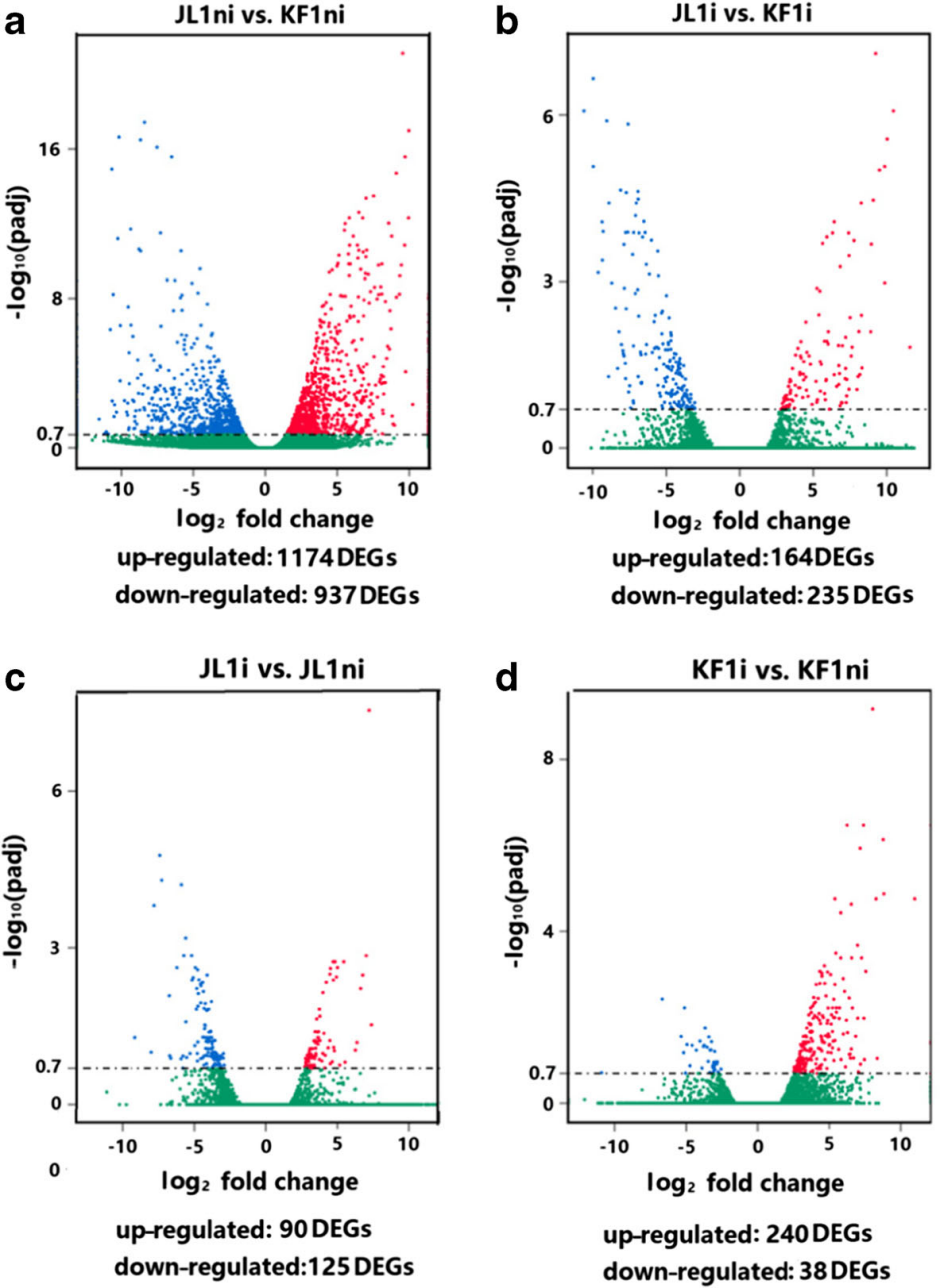
Transcriptional expression of genes in inoculated and non-inoculated leaves of HR/JL1 and HS/KF1*.* **a**: displaying the log_2_ fold change in expression of genes in JL1ni vs. KF1ni). **b**: differentially expressed genes (DEGs) in JL1i vs. KF1i). **c**: DEGs in JL1i vs. JL1ni. **d**: DEGs in KF1i vs. KF1ni. Y-axes indicates the mean of normalized counts (padj < 0.05) and X-axes indicates the log_2_ fold change values. DEGs are shown in blue, green and red indicating down-regulated, no change and up-regulated genes, respectively

In comparison of gene expression level in the non-inoculated samples (JL1ni vs. KF1ni), the expression level of 1174 and 937 DEGs were higher or lower in JL1 than that in KF1 (Fig. 2a), respectively, representing genotypic differences between HR and HS genotype. In the inoculated samples, the expression level of 164 genes were higher and 235 genes were lower in JL1 than that in KF1, implying these genes might be specifically involved in the process of soybean/*Pm* interaction (Fig. 2b).

**Fig. 3:**
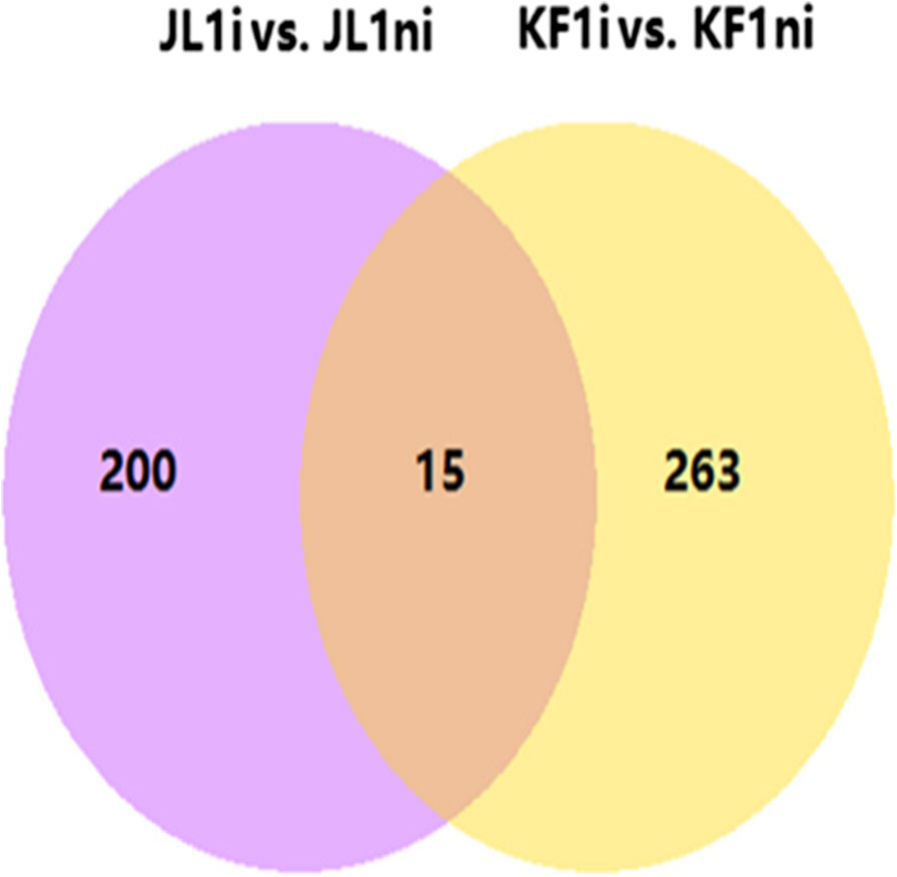
Venn diagram showing the number of DEGs identified 72 h after *Pm*-inoculation in the genotype of HR/JL1 and HS/KF1. The number of genes up- or down-regulated in the inoculated and non-inoculated samples (JL1i vs. JL1ni, KF1i vs. KF1ni) with a *P*-value < 0.001 and log_2_ fold change > 2.0 or < − 2.0 were drawn in diagram

In terms of paired comparison of up- and down-regulated DEGs in the inoculated vs. non-inoculated of HR or HS genotype, it was found that in the HR/JL1, out of 215 DEGs 90 were up-regulated and 125 were down-regulated (Figs. 2c and 3), while in the HS/KF1, of a total of 278 DEGs, 240 genes were up-regulated and 38 down-regulated, respectively (Figs. 2d and 3).

### Clustering of DEGs

**Fig. 4:**
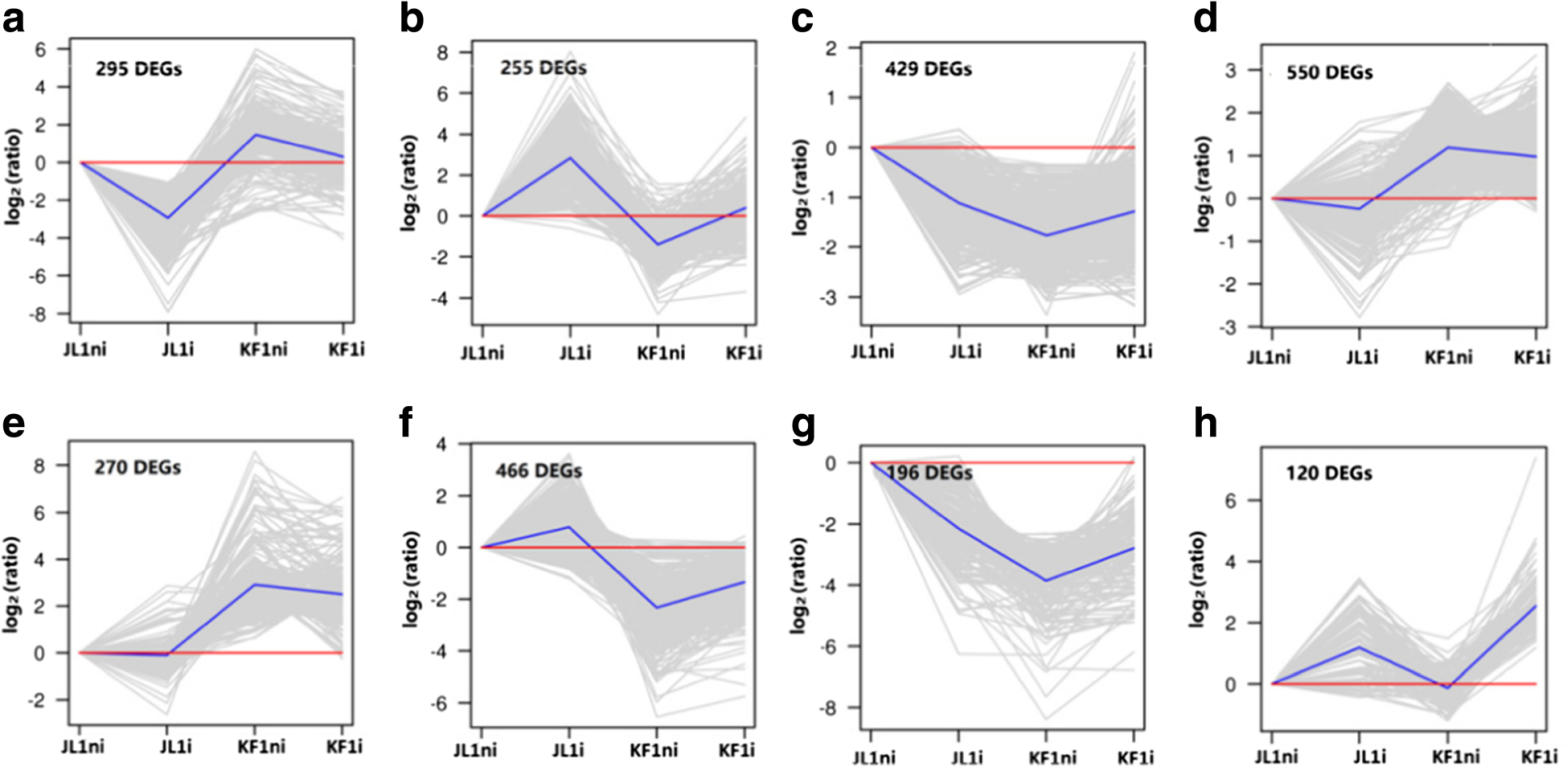
Clustering of 2581 detected DEGs. Genes were classified into eight subclusters **a**-**h** based on similarity of expression pattern. The gray lines represent the relative expression level of each gene within each sub-cluster from 4 libraries of JL1i, JL1ni, KF1i and KF1ni. The blue lines represent the mean of the relative expression level of all genes within each sub-cluster

H-clustering analysis was used to group 2581 DEGs into clusters and sub-clusters based on common expression patterns. Eight distinct sub-clusters A to H were generated reflecting the general trends and key transitional states in the HR and HS following *Pm*-inoculation. The number of genes within each cluster was presented in Fig. 4.

### Gene ontology (GO)

Based on the PFAM domain (http://pfam.xfam.org/), the 2581 DEGs were converted into GO-identities by mapping (http://www.geneontology.org). Mapped GO was then classified into three categories: biological process, cellular component and molecular function (Additional file 3: Table S3).

To understand the functional components involved in molecular responses to *Pm*, we annotated the most enriched GO terms in the HR/JL1 and HS/KF1 with significant difference at a level of corrected *P* value < 0.05 (Table 1).

**Table 1:** The most enriched GO terms in HR/JL1 and HS/KF1 with significant difference at a level of corrected *P* value < 0.05

GO terms	GO ID	Gene ID
JL1i vs. JL1ni Up-regulated		
Biological process		
Iron ion homeostasis	GO:0006879, GO:0055072, GO:0006826	Glyma01g31300, Glyma03g06420, Glyma07g19060, Glyma18g43650
Metal ion homeostasis	GO:0046916, GO:0055076, GO:0006875, GO:0055065, GO:0000041	
Cation homeostasis	GO:0030003, GO:0055080	
Ion homeostasis	GO:0006873, GO:0050801, GO:0098771	
Chemical homeostasis	GO:0048878, GO:0055082	
Molecular function		
Ferroxidase activity	GO:0004322	Glyma01g31300, Glyma07g19060, Glyma03g06420, Glyma18g43650
Oxidoreductase activity, oxidizing metal ions	GO:0016722, GO:0016724	
Ferric iron binding	GO:0008199	
Isocitrate lyase activity	GO:0004451	Glyma12g10780, Glyma06g45950
JL1i vs. JL1ni Down-regulated		
Biological process		
Photosynthesis	GO:0015979	Glyma09g08630, Glyma09g08260, Glyma10g32080, Glyma11g35130, Glyma12g34320, Glyma13g36240, Glyma15g01940
Photosynthesis, light harvesting	GO:0009765	Glyma09G08260, Glyma10g32080, Glyma11g35130
Oxazole or thiazole metabolic process	GO:0018131, GO:0046484, GO:0052837, GO:0052838	Glyma06g42080, Glyma20g27990
Cellular component		
Photosystem I & II	GO:0009521, GO:0009522, GO:0009523, GO:0034357	Glyma09g08260, Glyma09g08630, Glyma10g32080, Glyma11g35130, Glyma12g34320, Glyma13g36240, Glyma15g01940
Thylakoid	GO:0044436, GO:0009579	Glyma09g08260, Glyma09g08630, Glyma10g32080, Glyma11g35130, Glyma12g34320, Glyma13g36240, Glyma15g01940
Molecular function		
Chlorophyll binding	GO:0016168	Glyma09g08260, Glyma10g32080, Glyma11g35130
KF1i vs. KF1ni Up-regulated		
KF1i vs. KF1ni Down-regulated		
Biological process		
Photosynthesis	GO:0015979	Glyma03g42310, Glyma13g36240, Glyma13g43370, Glyma15g01940
Cellular component		
Photosystem I & II	GO:0009521, GO:0034357, GO:0009522, GO:0009538	Glyma03g42310, Glyma13g36240, Glyma13g43370, Glyma15g01940
Thylakoid	GO:0044436, GO:0009579	Glyma03g42310, Glyma13g43370, Glyma15g01940

It was notable that the DEGs related to photosystem were all down-regulated upon *Pm-*inoculation in both HR and HS genotypes. Under the category of biological process, 7 DEGs associated with photosynthesis and 2 DEGs related to thyazole metabolic process were down-regulated in the HR genotype. Similarly, in the category of cellular component, 7 enriched DEGs related to photosynthesis and 7 DEGs associated with thylakoid were down-regulated in HR genotype, while in the HS genotype 4 DEGs in the category of biological process and 4 DEGs in the cellular component were also down-regulated. This common feature reflects inhibition of photosystem in soybean plants upon *Pm*-infection.

In contrast to the reduction of photosynthesis, 5 of the most enriched DEGs under the category of biological process and 4 DEGs in the category of molecular function, that all associated with ion homeostasis (e.g. oxidizing metal ions, ferric iron binding, ferroxidase and oxidoreductase activity), were up-regulated in HR genotype, implicating modification of diverse ion channels after inoculation might play a vital role in defense responses to *Pm* invasion (Table 1).

### KEGG pathway analysis

In doing KEGG analysis, 2581 DEGs were annotated to KEGG pathway (http://www.genome.jp/kegg/), by which a total of 216 DEGs were identified. Results showed that in HR/JL1, among a total of 107 DEGs, there were 64 DEGs up-regulated involving in 31 KEGG pathways and 43 DEGs down-regulated participating in 19 different KEGG pathways (Additional file 4: Table S4). In HS/KF1, of 109 DEGs, 91 were up-regulated involving in 26 KEGG pathways and 18 were down-regulated involving in 13 KEGG pathways (Additional file 5: Table S5).

Apart from the basic metabolic pathways, e.g. carbohydrate, amino acid and lipid metabolism, the most interest to us were pathways participating phytohormone signalling transduction and plant/pathogen interactions based on the knowledge already published in the literature.

In the plant hormone signaling, two genes encoding either EFB1/2 (EIN3-binding F-box protein1-like, involving in ethylene signaling) or AUX/IAA protein (auxin-responsive protein IAA, involving in auxin signaling) were identified in JL1i vs. JL1ni (Table 2), while in KF1i vs. KF1ni two AUX/IAA genes and seven JAZ (JASMONATE ZIM-motif) genes (involving in jasmonate signaling), and one BAK1 protein kinase gene (Brassinosteroid insensitive1-associated receptor kinase1) were detected (Table 2). In addition, in the glyoxylate and dicarboxylate metabolism, two genes coding for isocitrate lyase, a key catalytic enzyme involving in ROS signaling, were identified. All of these results demonstrated that these genes were actively involving in the complex phytohormone signaling pathways.

**Table 2:** SDM defense responsive DEGs with *P* value < 0.001 and log_2_ fold change > 2 or < − 2

Terms	Gene ID(Glyma)	Read counts				Log2 fold change			
		KF1ni	KF1i	JL1ni	JL1i	KF1 vs. KF1ni	JL1ni vs. KF1ni	JL1i vs. KF1ni	JL1i vs. JL1ni
Plant hormone signal transduction	**11**								
AUX	**2**								
AUX/IAA	13g18910	115.33	968.76	213.16	2345.50	3.07	0.89	4.35	3.46
GH3	02g13910	43.49	373.79	84.81	398.11	3.10	0.96	3.19	2.23
Brassinosteroid	**1**								
BAK1	05g24770	108.11	1407.49	1045.10	560.86	3.70	3.27	2.38	−0.90
Ethylene	**1**								
EBF1	04g20330	32.24	586.80	184.34	2353.68	4.19	2.52	6.19	3.67
Jasmonate	**7**								
JAZ1	01g41290	78.44	1286.07	44.57	542.98	4.04	−0.82	2.79	3.61
	09g08290	150.06	7641.92	382.12	8135.14	5.67	1.35	5.76	4.41
	11g04130	24.65	631.92	1.67	315.76	4.68	−3.89	3.68	7.57
	13g17180	56.70	2397.55	73.33	3688.38	5.40	0.37	6.02	5.65
	15g19840	265.77	7262.01	432.97	10,025.92	4.78	0.70	5.24	4.53
	17g05540	7.67	306.83	19.98	222.05	5.32	1.38	4.86	3.47
JAZ6	07g04630	646.79	2896.85	524.87	3135.59	2.16	−0.30	2.28	2.16
Plant/pathogen interaction	**31**								
Oxidation	**8**								
Glyoxylase	07g03560	91.03	227.06	313.50	2652.58	1.32	1.78	4.87	3.08
Isocitrate lyase	06g45950	10.77	873.50	1262.32	9650.25	6.34	6.87	9.81	2.93
	12g10780	116.44	1546.11	1259.75	14,926.85	3.73	3.44	7.00	3.57
Peroxidase	01g32270	0.00	2.33	2.50	73.06	Inf^a^	Inf^a^	Inf^a^	4.87
	09g41440	1.55	120.58	4.75	227.35	7.04	1.62	7.20	5.58
Peroxidase 62-related	08g19180	1.03	451.44	4.23	364.86	8.77	2.04	8.47	6.43
	15g05810	0.52	67.97	1.21	5.65	7.04	1.22	3.44	2.22
	15g05820	74.82	3413.46	94.09	577.25	5.51	0.33	2.95	2.62
Calmodulin	**12**								
ACA4	03g31420	202.96	1875.99	760.97	4474.46	3.20	1.91	4.46	2.56
CML	10g32190	14.00	788.07	195.32	626.99	5.81	3.80	5.49	1.68
	10g00470	6.00	270.76	94.11	1064.77	5.50	3.97	7.47	3.50
CML24	18g04450	346.73	1903.08	30.25	301.65	2.46	−3.52	−0.20	3.32
CML44	13G09550	30.16	736.89	159.44	1104.95	4.61	2.40	5.20	2.80
CML1-related	05g13900	2618.57	36,645.18	4992.34	30,027.30	3.81	0.93	3.52	2.59
CML-like	03g39170	19.28	853.73	214.17	4922.86	5.47	3.47	8.00	4.52
	03g39180	57.14	1443.30	31.25	8369.36	4.66	−0.87	7.20	8.06
CML24-like	05g33880	172.33	3470.54	438.99	3944.01	4.33	1.35	4.52	3.17
	08g05810	149.12	12,677.75	239.19	13,111.02	6.41	0.68	6.46	5.78
CML44-like	14g24810	4.90	702.56	71.12	517.40	7.16	3.86	6.72	2.86
CML-dependent	11g13740	191.84	12.92	7.56	1.41	−3.89	−4.67	−7.10	−2.42
PR proteins	**11**								
PR proteins	01g31730	0.52	74.89	11.38	2174.33	7.18	4.45	12.03	7.59
	03g30420	3.22	305.06	507.98	1023.66	6.56	7.30	8.31	1.02
	06g40710	133.49	903.17	0.90	7.30	2.76	−7.22	−4.19	3.01
	16g06940	165.99	1996.03	834.75	2864.73	3.59	2.33	4.11	1.78
FLS2	08g08810	49.37	3483.31	19.60	613.61	6.14	−1.33	3.64	4.97
	08g08781	2.19	161.18	0.00	26.67	6.20	0.00	3.61	Inf^a^
LOX1	13g31280	136.67	1071.67	68.13	87.54	2.97	−1.00	−0.64	0.36
RPW8	01g32825	12.50	473.23	5.45	60.99	5.24	−1.20	2.29	3.49
PEPR1	10g33970	13.48	321.96	38.23	693.94	4.58	1.50	5.69	4.19
PEPR1/2	12g00960	66.44	797.47	340.30	710.24	3.59	2.36	3.42	1.06
	13g32630	17.61	367.59	348.28	1724.20	4.38	4.31	6.61	2.31
Transcription factors	**10**								
bHLH	**1**								
bHLH103	15g07010	27.74	354.74	71.50	993.14	3.68	1.37	5.16	3.80
MYB	**6**								
MYB-related	09g29800	216.98	1442.26	121.66	1074.53	2.73	−0.83	2.31	3.14
	10g05560	39.98	288.14	20.12	270.80	2.85	−0.99	2.76	3.75
MYB32- related	02g41180	36.70	93.29	72.15	685.06	1.35	0.98	4.22	3.25
MYB-related TF LHY	03g42260	183.04	981.94	69.66	598.94	2.42	−1.39	1.71	3.10
	19g45030	215.40	1042.06	125.26	1205.87	2.27	−0.78	2.49	3.27
MYB-like	03g34110	15.35	189.20	68.03	911.41	3.62	2.15	5.89	3.74
WRKY	**3**								
WRKY4	01g31921	43.63	765.05	139.06	2145.04	4.13	1.67	5.62	3.95
WRKY31	04g06495	4.12	145.38	103.06	341.37	5.14	4.65	6.37	1.73
WRKY156	17g04710	7.48	154.07	138.48	52.69	4.36	4.21	2.82	−1.39

inf^a^ represents infinitive fold change

Bold data represent the total number of DEGs belong to different terms

In the plant pathogen interaction, three genes in JL1i vs. JL1ni were detected including genes coding for LRR receptor-like serine/threonine-protein kinase FLS2 protein (Flagellin-sensitive 2), calcium-binding protein CML, and a protein associated with cyclic nucleotide gated channel (Table 2). In KFi vs. KF1ni, totally 18 genes were identified, including seven JAZ genes, seven calmodulin genes, one BAK1, one DNA-binding WRKY (Glyma01g31921), and one FLS2 (Table 2).

Except one calmodulin gene was down-regulated, all above mentioned DEGs in the pathways of phytohormone signal transduction and plant/pathogen interaction were up-regulated in both genotypes of HR and HS after inoculation with *Pm* (Table 2).

### SDM defense responsive genes

In searching SDM defense responsive genes, a total of 52 DEGs either up- or down-regulated were identified. Of the total, 31, 11 and 10 DEGs were categorized into phytohormone signalling transduction, plant/pathogen interaction and TFs, respectively (Table 2).

In the phytohormone signalling pathway, 12, 8, 7, 1, 1 and 2 DEGs participating calmodulin, SA/ROS, JA, ET, BR (brassinosteroid) and AUX (auxin), respectively, in response to *Pm*-infection. In particular, 3 and 4 peroxidase genes were highly expressed with 4–6-fold increase in HR/JL1 and 7–9-fold increase in HS/KF1 by comparison of inoculated with non-inoculated samples. In the category of plant/pathogen interaction, 11 PR proteins were involved. In terms of TFs, 1 bHLH, 6 MYBs and 3 WRKYs were identified in the study (Table 2).

By comparison of the expression level of the 52 DEGs in non-inoculated samples of JL1ni and KF1ni, if the read counts of KFni was taken as 1, then the log_2_ fold change of 38 DEGs in JL1ni were higher than that in KF1ni, accounting for 73% of the total DEGs. Noteworthy, there were 14 DEGs associated with plant/pathogen interaction (including 5 PR proteins, 5 calmodulins and 4 oxidative proteins) and 3 transcription factors, all of which with log_2_ fold change value greater than 2.0, demonstrating that the basal expression level of these DEGs were much higher in HR genotype without fungal pathogen and might be served as basal defense responsive genes (Table 2).

On the other hand, when comparing the data of HR and HS after fungal inoculation, it was found that 50 DEGs in KF1i were up-regulated with log_2_ fold change all greater than 2,0, accounting for 96% of the total. While in the JL1i, 43 DEGs (83%) with log_2_ fold change greater than 2,0 were found after pathogen attack. These data suggested that the HS responded to *Pm* infection more quickly and strongly. By counting the number of DEGs, however, it was found that the read counts of 30 DEGs in JL1i were still higher than that in KF1i (Table 2).

### qRT-PCR validation

To validate the RNA-seq expression data and its reliability, seven DEGs were randomly selected for Real-time quantitative PCR (qRT-PCR) analysis. To compare these two methods, the relative expression measurement from qRT-PCR was transformed into fold change by base 2 to match with the RNA-seq fold change value. The 7 selected genes for this comparison included five defense responsive genes i.e. Glyma08g08810, Glyma04g20330, Glyma04g10940, Glyma16g06940 and Glyma06g40710 and two WRKYs. Results of comparison between qRT-PCR and RNA-seq showed similar expression patterns indicating they were correlated. Therefore, the RNA-seq data could be used for gene expression profiling of soybean in response to *Pm*-inoculation. The primer set used for qRT-PCR were listed in Additional file 6: Table S6.

## Discussion

The sequences of soybean genome publicly available at Phytozome (http://www.phytozome.net/soybean) coupled with availability of next-generation RNA sequencing analysis provided a powerful tool to study the differential expression of genes in soybean plants in response to biotic and abiotic stresses. Kim et al. (2011) reported the first application of RNA-seq for profiling gene expression in soybean in response to pathogen attack. In their study, the transcriptomes of two near isogenic lines (NILs), one resistant and one susceptible to bacterial leaf pustule (*Xanthomonas axonopodis* pv. *glycines*), were analyzed 0, 6, and 12 hai and a total of 2761 DEGs, including a set of defense response genes, were identified. In the following years, transcriptomic analyses were reported on Asian soybean rust (*Phakopsora pachyrhizi*) [11], soybean root and stem rot (*Phytophthora sojae*) [10] in soybean, and soybean cyst nematode (SCN; *Heterodera glycines*) in common bean [12]. By which the responsive genes and metabolism pathways associated with disease resistance have been identified. Unfortunately, no such a study in soybean/*Pm* interaction has been reported so far. Here, we provided data demonstrating there were significant transcriptomic variations in SDM-resistant and susceptible genotypes of soybean in response to *Pm*-infection.

### SDM resistance and inoculation

In our previous study, we cloned a *GmSAGT*1 gene from a HR genotype of soybean, Zaofeng5. After 4 h of SA induction, the transcript level of this gene in the HR genotype was 13 times higher than that in HS genotype Heinong10, demonstrating that higher expression of *GmSAGT*1 gene was associated with increased SDM resistance [7]. In the current study, we used an additional pair of genotypes i.e. JL1 (HR) and KF1 (HS) as plant materials for conducting transcriptomic analysis. JL1 was bred by the Soybean Institute, Jilin Academy of Agricultural Sciences in 1979. This cultivar was derived from a cross between cultivated soybean (*G. max*) and wild spices (*G. sojae*, GD50477). It featured as small grain size, relative narrow and long leaves, early maturity and higher resistance to SDM. These characters might be derived from wild species of soybean [13, 14]. The KF1 was a commercial variety in late 1990s with SMV (soybean mosaic virus) resistance that later on turned to be highly susceptible to SDM.

For understanding differential expression of genes in response to *Pm* infection, it is important to know the sampling time point corresponding to what stage of pathogen invasion. In this study, 72 hai was used. According to the literature, the 72 hai was corresponding to the stage of hyphae growth and haustoria formation of *Pm* fungi in the soybean plant tissue. Riggle (1974) conducted a detailed histopathological study of *Pm* on leaves of soybean cultivars with varying degree of resistance and on a non-host. It indicated that the germination of conidia, germ tube growth, appressorium formation, development of penetration pegs, and hyphal growth for 36 hai were about the same on all categories of plants, including a susceptible, a resistant near isogenic line (NIL), a heterozygous line for resistance, a moderately resistant cultivar and a non-host common bean. During 36 to 120 hai, hyphae grew rapidly on the leaves of susceptible cultivars and the number of haustoria/mm of hyphae was high. However, on the resistant NIL, the hyphae growth was very slow and few haustoria were formed. The author concluded that the resistance appeared to be related to slow rate of hyphal growth and the ability of hyphae to form haustoria. These data suggested that the 72 hai used in the current study was the right time point to represent the early stage of *Pm* invasion. The DEGs identified in the HR and HS genotype at this stage might provide insight and expand our knowledge in understanding the molecular basis of SDM resistance.

### SDM defense responsive genes

By comparison of the read counts of the 52 SDM-defense responsive DEGs, it was interesting to find that the expression levels of most DEGs (73%) in HR non-inoculated samples were higher than that in the HS non-inoculated ones (Table 2). It indicated that the basal expression levels of these DEGs might be sufficient for resistance and could be serve as basal defense responsive genes. In contrast, by comparison of the log_2_ fold change value of inoculated vs non-inoculated samples of HR and HS, it showed that the susceptible KF1 reacted to *Pm* attack more rapidly and strongly (Table 2). This was not surprising since SDM lesions were developed on leaves of HS plant 72 hai, while during this period only few hypersensitive spots showed on leaves of HR plant. Although HS/KF1 responded to fungal attack more quickly, the read counts of DEGs (30/52, 58%) were still lower than that in HR/JL1 after inoculation (Table 2). It should also note that, after *Pm* inoculation, the log2 fold change value of 83% DEGs in HR and 96% DEGs in HS were greater than 2.0 (Table 2), demonstrating additive effects existed following fungal invasion.

Plants have developed two defense mechanisms in its evolution, which are (i) pathogen triggered immunity (PTI) that confers non-host resistance by recognizing a broad range of pathogens with conserved molecular pattern. (ii) effectors-triggered immunity (ETI), in which the pathogen effectors are recognized by specific R genes that encode nucleotide binding site and NBS-LRR domains [15, 16]. Unfortunately, to the best of our knowledge, the ETI genes specifically resistant to *Pm* have not yet been reported to date in soybean due to both genetic and pathogenic analysis at molecular level are lacking. As such, the gene-for-gene interaction between soybean and *Pm* is unclear so that it is not possible to classify the putative genes detected in this study into PTI and ETI. Therefore, we further analyzed the genes involving in phytohormone signalling pathways and the genes encoding PR proteins as well as TFs based on the existing knowledge and the functional annotation of DEGs in GO and KEGG analysis in the study.

It has been shown that SA, JA and ET play major roles in response to biotic stress as their levels increase with pathogen infection [17, 18]. Upon pathogen infection, the early signalling events occurred, including increase of intracellular Ca^2+^ concentration and production of secondary signalling molecules such as ROS. In our GO analysis, 5 of the most enriched DEGs (iron ion homeostasis, metal ion homeostasis, cation homeostasis, ion homeostasis, chemical homeostasis) under the category of biological process were all associated with ion homeostasis and 4 DEGs (ferroxidase activity, oxidoreductase activity, ferric iron binding, isocitrate lyase activity) in the category of molecular function were associated with oxidation as well as ROS production (Table 1). In the analysis of SDM defense responsive genes, 10 DEGs coding for calmodulin proteins and 1 coding for calmodulin-regulated Ca^2+^ ATPase (ACA4) were identified (Table 2), indicating a wide range of genes participated in the modulation of Ca^2+^ metabolism.

Studies have shown that Ca^2+^ functions in concert with other important second messengers like ROS [19]. In the SA signalling pathway, a rapid increase in the rate of ROS production, known as ‘the oxidative burst’, occurs in response to biotic and abiotic stresses [20]. Higher rate of ROS production results in the induction of PR genes expression which in turn leading to a long-lasting and broad-spectrum induced resistance known as systemic acquired resistance (SAR) [18]. Once SA pathway is activated, the expression of downstream PR genes is induced. Results obtained in this study indicated there were four genes encoding PR proteins (Glyma01g31730, Glyma03g30420, Glyma06g40710, Glyma16g06940) that were highly differential expressed (Table 2).

In soybean, our previous study indicated that after SA induction, the peroxisome-located gene *GmSAGT*1 and its upstream gene *GmGOX* coding for glycolate oxidase were highly expressed in soybean resistant variety than that in susceptible ones [7]. The function of GmGOX protein is to convert glycolic acid into glyoxylate and H_2_O_2_ by oxidation [21]. Interestingly, in the current study we have identified seven DEGs coding for oxidative enzymes, including one glyoxylase, two isocitrate lyases and four peroxidases. It is known the isocitrate lyase is a key enzyme in the glyoxylic acid cycle, which catalyzes the lysis of isocitric acid to produce succinic acid and glyoxylic acid. Subsequently, the resultant glyoxylic acid is further converted to serine and H_2_O_2_. These findings have provided evidence that SA in conjunction with ROS appear to be involved in the establishment of systemic defenses and play an essential role in H_2_O_2_ accumulation, PR gene expression, hypersensitive response and SAR induction [22].

**Fig. 5:**
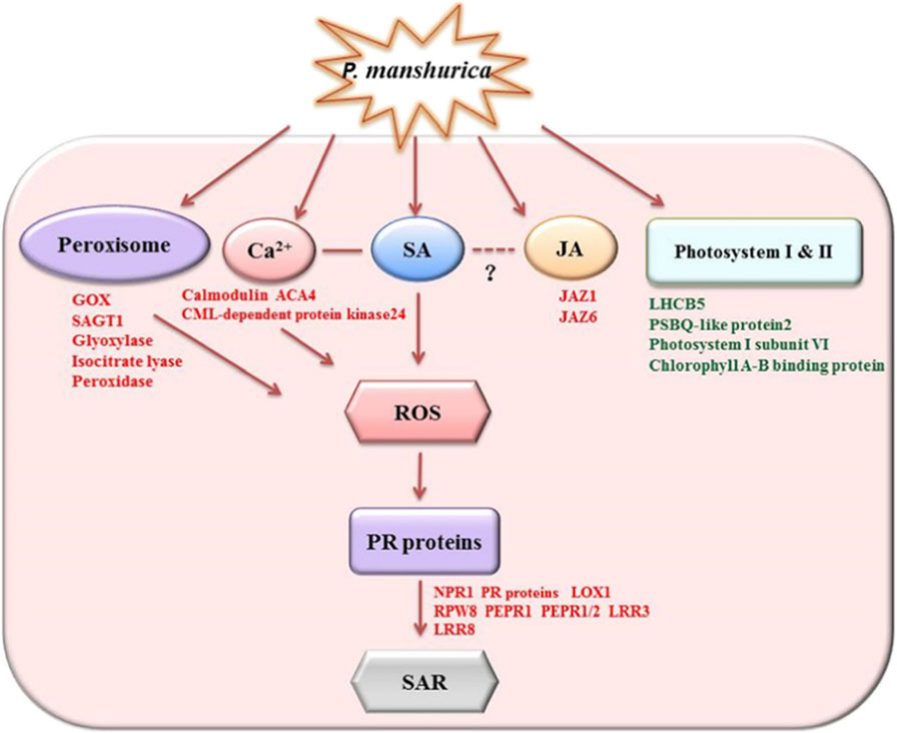
A schematic diagram of soybean/*Pm* interaction showing the molecular processes and the involvement of DEGs identified in this study. Note: the DEGs with red color are up-regulated while the DEGs in green are down-regulated

SA is generally involved in the activation of defense responses against biotrophic and hemi-biotrophic pathogens [23], whereas, JA and ET are responsible for defense against necrotrophic pathogens [24, 25]. The *P. manshurica* is a biotrophic pathogen and the annotation of the most DEGs identified in this study let us reasonably to suggest SA signalling coupled with ROS is a key prominent pathway in soybean in response to *Pm* infection. Based on above results, a schematic diagram was drawn in describing the molecular processes and the involvement of DEGs in soybean host upon *Pm*-invasion (Fig. 5).

Recent studies have shown that there is sophisticated crosstalk between different hormone-mediated pathways. In a recent review article, Verma et al. (2016) have emphasized that the defense responses activated in plants in response to different stresses depends on the type of crosstalk (positive or negative) between the hormone signaling pathways rather than solely on the individual contributions of each hormone.

It is commonly viewed that SA and JA regulate biotic stress responses antagonistically [17]. Studies have shown NPR1 to be a key player in the antagonistic crosstalk of SA and JA [26]. The SA-facilitated suppression of JA-responsive genes like LIPOXYGENASE 2 (LOX2) has been reported [27]. Interestingly, an isozyme LOX1 was also detected in the present study, however, it was up-regulated after *Pm* inoculation. Moreover, it is note-worthy that, in contrast to antagonistic crosstalk between SA and JA, we found they are simultaneously activated since seven JAZ and two FLS2 were highly differential expressed in both HR/JL1 and HS/KF1 after *Pm* infection. Although most studies prove antagonistic interaction between SA and JA, synergistic interactions have also been observed at low SA-JA concentrations and by simultaneous induction of both defenses [28, 29].

The DEGs involving in ET, BR and AUX signalling pathways were detected in this study. It was reported that JA and ET operate synergistically in regulating defense related genes after pathogen infection [30]. However, in considering JA and ET are responsible for defense against necrotrophic pathogens [30] and auxin is mainly responsible for plant growth and development [18], the roles of these signalling in SDM defense responses require further elucidation. Due to the complexity of crosstalk between different plant hormones and in line with the simultaneous activation of SA and JA observed in this study, it is of great interest to investigate how they crosstalk one another and whether they are synergistically reacted. All of these remain to be addressed.

### Transcription factors

In the study, three types of TFs including one bHLH (the core JA-signalling components MYC2), six MYBs and three WRKYs, which differentially expressed in HR/JL1i vs. JL1ni and HS/KF1i vs. KF1ni, were identified (Table 2).

MYC2 is a basic helix-loop-helix (bHLH) TF that recognizes the G-box and G-box variants in the promoter of its target genes and regulates different branches of the JA pathway [31–37]. MYB1 gene in tobacco is known to encode a signaling component downstream of SA that may participate in transcriptional activation of PR genes and plant disease resistance [38]. MYBs and WRKYs are large families in plant genome, in soybean there are 252 and 188 members of MYB [39] and WRKY families [40], respectively.

WRKY proteins, a large family of transcriptional regulators that has to date only been found in plants. Many previous studies have shown WRKYs are involved in the plant response to biotic and abiotic stresses either act as transcriptional activators or repressors [41–43]. In soybean, considerable effort has gone into identifying WRKY TFs and its mediated gene expression in response to pathogen invasion. Bencke-Malato et al. [11] have conducted a detailed study on genome-wide annotation of the soybean WRKY family and functional characterization of genes involved in response to *Phakopsora pachyrhizi* (Asian soybean rust) infection. In this study, we have identified three WRKYs, i.e. *GmWRKY*4 (Glyma01g31921), 31 (Glyma04g06495) and 156 (Glyma17g04710) (the name of *GmWRKYs* are given according to the Yu et al., [40]), that are highly differential expressed following *Pm* inoculation. In particular, their expression level has increased 4.13- to 5.14-fold in HS/KF1, while it has increased 1.39- to 3.95-fold in HR/JL1. To date, the roles of WRKYs in soybean immune response to different pathogen attack and how WRKYs react with their target genes or crosstalk to the genes involving in the SA-, JA- and other plant hormone signalling remain largely unknown.

## Conclusion

Comparative transcriptomic analysis of HR and HS genotype with and without inoculation allowed us to explore defense responsive genes to *P. manshurica* infection. Totally, 52 DEGs were identified in the study, which participated in the SA/ROS and JA signalling pathway resulting in the calcium modulation, oxidative burst and PR protein production. Of particular interest are the DEGs coding for glyoxylase, isocitrate lyase and an array of peroxidase. The PR proteins and TFs are also worth to be further investigated and characterized to identify candidate genes resistant to soybean downy mildew disease. As plant defense response is a very complex system, which needs further comprehensive analysis step by step.

## Methods

### Plant materials and *Pm*-infection

In our previous study [7], 15 soybean genotypes were classified into five categories: HR (highly resistant), R (resistant), MR (moderate resistant), S (susceptible) and HS (highly susceptible) by using in vitro leaf/*Pm* inoculation or by field observation. In the current study, we have used JL1 (HR) and KF1 (HS) as plant materials for conducting transcriptomic analysis.

To prepare source of *Pm,* infected leaves (mixture of different races) were collected from soybean field at Shenyang Agricultural University. Collected leaves were placed on moisture paper in a Petri dish and maintained at 20 °C overnight for proliferation of conidia. Leaves were then washed with autoclaved double distilled water to collect conidia followed by dilution to a final concentration of 5×10^5^ conidia/ml.

Back side of the top third expanded leaves of 40 d-old seedlings were inoculated using brush pen. Leaves of JL1 and KF1 brushed with distilled water in the same manner without pathogen were used as a control. Plants were then placed in a growth chamber under conditions of 20/24 °C, 14 h dark/10 h light cycle and relative humidity ≥95%.

### Sample preparation and RNA sequencing

Total RNA was extracted from leaves 72 hai using RNAprep Pure Plant Kit (Tiangene Co., China) following the manufacturer’s instructions. To prepare cDNA samples for transcriptome sequencing, the quantity of total RNA was assessed with Nanodrop spectrophotometer (Thermo Fisher Scientific Inc., USA). The RNA quality and integrity was evaluated on the Agilent Bioanalyzer 2100 system (Agilent Technologies, USA) and then processed with NEBNext Ultra RNA Library Prep Kit. The cDNA fragments of ~ 150 bp in length were selected and further enriched with PCR amplification to generate cDNA libraries. Four cDNA libraries derived from JL1ni, JL1i, KF1ni and KF1i, each with two biological replicates, were used for Illumina sequencing. RNA-sequencing was conducted by Allwegene Co., China using equipment of HiSeqTM2000/MiSeq, which were technically repeated three times.

### Reads filtering, alignment and de novo assembly

Before assembly, the raw reads were first filtered to gain high quality clean reads by removing low quality reads as well as reads containing adaptor or unknown nucleotides larger than 10%. Clean reads were de novo assembled into transcripts based on the soybean reference genome sequence. For removing redundancy, when a component contained more than one assembled transcript, only the longest one was preserved. The clean reads from each RNA-seq library were aligned with soybean genome (*Glycine max* Wm82.a2.v1) as a reference (http://www.phytozome.net/soybean) using TopHat software (http://tophat.cbcb.umd.edu). Resulted read counts for each matched gene were normalized with RPKM (reads per kilo bases per million reads) to measure its expression level. For gene annotation, the matched genes were searched by BLAST against the databases of Phytozome and NCBI (www.ncbi.nlm.nih.gov/).

### Differential expression of genes in response to *Pm* infection

Genes differentially expressed in JL1i vs. JL1ni and KF1i vs. KF1ni with *P*-value < 0.001 were selected and the difference of expression level was log_2_-transformed and filtered at a level of 2-fold or greater. These genes were considered as DEGs. DEGs in inoculated and non-inoculated leaves of HR and HS genotype were represented as Venn diagrams.

Annotation of the up- and down-regulated genes was carried out based on the identification of conserved PFAM domains (pfam.xfam.org). PFAM domains were converted into GO-identities by mapping to GO (http://www.geneontology.org).

DEGs were further clustered according to the expression patterns using H-cluster method. The significantly enriched GO terms involving in biological process, cellular component and molecular function in response to soybean/*Pm* interaction were identified. In addition, the DEGs were mapped to soybean KEGG database using the DEGseq package [44] to search the genes involving in different KEGG pathways. To identify genes in soybean immune responses to *Pm* infection, the up- and down-regulated DEGs and TFs were analyzed based on annotation of their function.

### Validation of RNA-seq data by qRT-PCR

Seven DEGs were randomly selected for qRT-PCR analysis for validation of RNA-seq data. In brief, 1.0 μg of total RNA prepared from each sample was first reverse transcribed into its cDNA with oligo(dT)18 through M-MLV Reverse Transcriptase (TaKaRa, Japan). The qPCR was then performed on an ABI 7500 Real-Time System (Applied Biosystems, USA) with a 20 μl reaction system. In which it contained 2 μl of cDNA, 0.8 μl of each primer, 6 μl of sterile water, 10 μl of SYBR premix TagII (TaKaRa, Japan), and 0.4 μl of Rox Reference DyeII (TaKaRa, Japan). The conditions for PCR amplification were: 15 s denaturation at 95°C followed by 40 cycles at 95 °C for 5 s, 60 °C for 34 s, and 72 °C for 10 s. Primers used for qRT-PCR were designed by DNAMAN software and listed as Additional file 6: Table S6.

Soybean *Actin* gene (Glyma 08g19420) was used as an internal control. Samples were analyzed based on the stable expression level of the reference gene according to the method described by Livak & Schmittgen [45]. Three independent replicates were performed for each sample. The data of mean and standard deviation were calculated and statistically analyzed by Student’s T-test.

## Additional files

Additional file 1: **Table S1.** Overview of the sequencing reads. Note: a adaptors and low-quality reads were excluded. b Q20: The percentage of bases with quality value larger than 20. c Q30: The percentage of bases with quality value larger than 30. JL1: HR genotype. KF1: HS genotype. i: inoculated. ni: non-inoculated. (DOCX 18 kb)

Additional file 2: **Table S2.** Statistics of clean reads mapped to soybean reference genome. Note: JL1: HR genotype. KF1: HS genotype. i: inoculated. ni: non-inoculated. (DOCX 17 kb)

Additional file 3: **Table S3.** GO terms identified in soybean HR (JL1i vs. JL1ni) and HS (KF1i vs. KF1ni). Note: A total of 2581 genes were mapped and categorized to GO terms. Data show the number of genes that were up- or down-regulated within the categories of biological process, cellular component and molecular function. The * represents DEGs with significant difference at a level of corrected *P* value < 0.05. (DOCX 23 kb)

Additional file 4: **Table S4.** KEGG classification of the differentially expressed genes in HR/JL1. Note: Up- and down-regulated NCBI-Gene IDs are non-underlined and underlined, respectively. (DOCX 19 kb)

Additional file 5: **Table S5.** KEGG classification of the differentially expressed genes in the HS/KF1. Note: Up- and down-regulated NCBI-Gene IDs are non-underlined and underlined, respectively. (DOCX 19 kb)

Additional file 6: **Table S6.** Primers used for qRT-PCR amplification of seven DEGs. Note: F: forward primers; R: reverse primers. (DOCX 17 kb)

## Abbreviations

ACA4: Calmodulin-regulated Ca^2+^-ATPase
AUX: Auxin
bHLH: Basic helix-loop-helix
BR: Brassinosteroid
DEGs: Differentially expressed genes
EFB1/2: EIN3-binding F-box protein1-like
ETI: Effectors-triggered immunity
FLS2: Flagellin sensing 2
GmSAGT: Serine/alanine glyoxylate amino transferase
GO: Gene ontology
GOX: Glycolate oxidase
HR: Highly resistant
HS: Highly susceptible
IAA: Indole-3-acetic acid
JA: Jasmonic acid
JAZ: JASMONATE ZIM-motif
KEGG: Kyoto encyclopedia of genes and genomes
LOX: Lipoxygenase
LRR: Leucine-rich repeat
NBS-LRR: Nucleotide binding site-leucine rich repeat
NIL: Near isogenic line
*Pm*: *Peronospora manshurica*
PR protein: Pathogenesis-related protein
PTI: Pathogen triggered immunity
qRT-PCR: Real-time quantitative PCR
ROS: Reactive oxygen species
SA: Salicylic acid
SAR: Systemic acquired resistance
SDM: Soybean downy mildew
TF: Transcription factor
