# Supplementary material for: Transcriptomic analysis of genes in soybean in response to Peronospora manshurica infection
Source: BMC Genomics. 2018 May 18;19:366. doi: 10.1186/s12864-018-4741-7 (PMC5960119; doi:10.1186/s12864-018-4741-7)
Supplement: Supplementary file 1 — Table S1. Overview of the sequencing reads. Note: a adaptors and low-quality reads were excluded. b Q20: The percentage of bases with quality value larger than 20. c Q30: The percentage of bases with quality value larger than 30. JL1: HR genotype. KF1: HS genotype. i: inoculated. ni: non-inoculated. (DOCX 18 kb) [file 12864_2018_4741_MOESM1_ESM.docx]

**Table S1 Overview of the sequencing reads.**

| Sample | Raw reads | Clean reads^a^ | Clean bases | Error (%) | Q20^b^ (%) | Q30^c^ (%) | GC (%) |
| --- | --- | --- | --- | --- | --- | --- | --- |
| JL1i | 32,211,688 | 30,096,880 | 4.52 G | 0.02 | 98.18 | 95.28 | 44.66 |
| JL1ni | 34,441,809 | 32,431,866 | 4.86 G | 0.02 | 98.15 | 95.24 | 44.54 |
| KF1i | 32,113,766 | 30,246,417 | 4.54 G | 0.02 | 98.11 | 95.14 | 44.72 |
| KF1ni | 29,449,407 | 27,298,156 | 4.10 G | 0.02 | 98.15 | 95.22 | 45.41 |
| Total | 128,216,670 | 120,073,319 | 18.02 G | --- | --- | --- | --- |

**Note:** ^a^ adaptors and low-quality reads were excluded. ^b^ Q20: The percentage of bases with quality value larger than 20. ^c^ Q30: The percentage of bases with quality value larger than 30. JL1: HR genotype. KF1: HS genotype. i: inoculated. ni: non-inoculated.
