# Supplementary material for: Transcriptomic analysis of genes in soybean in response to Peronospora manshurica infection
Source: BMC Genomics. 2018 May 18;19:366. doi: 10.1186/s12864-018-4741-7 (PMC5960119; doi:10.1186/s12864-018-4741-7)
Supplement: Supplementary file 2 — Table S2. Statistics of clean reads mapped to soybean reference genome. Note: JL1: HR genotype. KF1: HS genotype. i: inoculated. ni: non-inoculated. (DOCX 17 kb) [file 12864_2018_4741_MOESM2_ESM.docx]

**Table S2 Statistics of clean reads mapped to soybean reference genome.**

| Sample | Total clean reads | Mapped reads | Uniquely mapped reads | Multiple mapped reads |
| --- | --- | --- | --- | --- |
| JL1i | 30,096,880 | 24,373,032 (80.98%) | 23,936,022 (79.53%) | 437,011 (1.45%) |
| JL1ni | 32,431,866 | 26,119,050 (80.54%) | 26,119,050 (80.54%) | 417,354 (1.29%) |
| KF1i | 30,246,417 | 24,5547,17 (81.18%) | 24,151,871 (79.85%) | 402,846 (1.33%) |
| KF1ni | 27,298,156 | 21,952,273 (80.42%) | 21,538,541 (78.90%) | 413,732 (1.52%) |
| Total | 120,073,319 | 96,999,072 (80.78%) | 95,745,483 (79.47%) | 1,670,943 (1.39%) |

**Note:** JL1: HR genotype. KF1: HS genotype. i: inoculated. ni: non-inoculated.
