# Supplementary material for: Transcriptomic analysis of genes in soybean in response to Peronospora manshurica infection
Source: BMC Genomics. 2018 May 18;19:366. doi: 10.1186/s12864-018-4741-7 (PMC5960119; doi:10.1186/s12864-018-4741-7)
Supplement: Supplementary file 3 — Table S3. GO terms identified in soybean HR (JL1i vs. JL1ni) and HS (KF1i vs. KF1ni). Note: A total of 2581 genes were mapped and categorized to GO terms. Data show the number of genes that were up- or down-regulated within the categories of biological process, cellular component and molecular function. The * represents DEGs with significant difference at a level of corrected P value < 0.05. (DOCX 23 kb) [file 12864_2018_4741_MOESM3_ESM.docx]

**Table S3 GO terms identified in soybean HR (JL1i vs. JL1ni) and HS (KF1i vs. KF1ni)**

| GO terms | JL1i vs. JL1ni | | | | KF1i vs. KF1ni | | | |
| --- | --- | --- | --- | --- | --- | --- | --- | --- |
|  | **Up-regulated** | | **Down-regulated** | | **Up-regulated** | | **Down-regulated** | |
|  | **No.** | **%** | **No.** | **%** | **No.** | **%** | **No.** | **%** |
| Biological process |  |  |  |  |  |  |  |  |
| Photosynthesis & photoinhibition | 5 | 0.47 | 13* | 1.48 | 0 | 0.00 | 6* | 4.65 |
| Biological regulation | 107 | 10.16 | 167 | 18.96 | 604 | 22.17 | 21 | 16.28 |
| Cellular metabolic process | 369 | 35.04 | 376 | 42.68 | 1115 | 40.93 | 49 | 37.98 |
| Response to stimulus & signaling | 42 | 3.99 | 16 | 1.82 | 169 | 6.20 | 0 | 0.00 |
| Cellular transport | 47 | 4.46 | 27 | 3.06 | 12 | 0.44 | 0 | 0.00 |
| Developmental process | 64 | 6.08 | 57 | 6.47 | 162 | 5.95 | 17 | 13.18 |
| Multicellular organismal process | 136 | 12.92 | 67 | 7.60 | 97 | 3.56 | 4 | 3.10 |
| Ion homeostasis and transport | 93* | 8.83 | 5 | 0.57 | 110 | 4.04 | 14 | 10.85 |
| Protein metabolism | 51 | 4.84 | 51 | 5.79 | 111 | 4.07 | 6 | 4.65 |
| Cellular biosynthetic process | 139 | 13.20 | 102 | 11.58 | 344 | 12.63 | 12 | 9.30 |
| Subtotal | 1053 | 100.00 | 881 | 100.00 | 2724 | 100.00 | 129 | 100.00 |
| Cellular component |  |  |  |  |  |  |  |  |
| Cell part | 70 | 53.85 | 101 | 34.35 | 181 | 40.31 | 27 | 30.34 |
| Cell structure | 1 | 0.77 | 18 | 6.12 | 8 | 1.78 | 1 | 1.12 |
| Extracellular region | 6 | 4.62 | 2 | 0.68 | 7 | 1.56 | 4 | 4.49 |
| Intracellular | 0 | 0.00 | 14 | 4.76 | 27 | 6.01 | 4 | 4.49 |
| Macromolecular complex | 0 | 0.00 | 34 | 11.56 | 17 | 3.79 | 11 | 12.36 |
| Membrane system | 38 | 29.23 | 30 | 10.20 | 99 | 22.05 | 4 | 4.49 |
| Organelle | 15 | 11.54 | 50* | 17.01 | 110 | 24.50 | 23* | 25.84 |
| Photosystem | 0 | 0.00 | 45* | 15.31 | 0 | 0.00 | 15* | 16.85 |
| Subtotal | 130 | 100.00 | 294 | 100.00 | 449 | 100.00 | 89 | 100.00 |
| Molecular function |  |  |  |  |  |  |  |  |
| Antioxidant activity | 2 | 0.35 | 0 | 0.00 | 4 | 0.21 | 0 | 0.00 |
| Antiporter activity | 7 | 1.23 | 0 | 0.00 | 6 | 0.32 | 0 | 0.00 |
| Binding | 281 | 49.38 | 393 | 55.98 | 1098 | 58.22 | 107* | 62.21 |
| Catalytic activity | 37 | 6.50 | 41 | 5.84 | 80 | 4.24 | 9 | 5.23 |
| Channel activity | 3 | 0.53 | 10 | 1.42 | 10 | 0.53 | 0 | 0.00 |
| Electron carrier activity | 0 | 0.00 | 2 | 0.28 | 10 | 0.53 | 1 | 0.58 |
| Enzyme regulator activity | 142* | 24.96 | 151 | 21.51 | 396 | 21.00 | 22 | 12.79 |
| Molecular transducer activity | 62 | 10.90 | 79 | 11.25 | 165 | 8.75 | 0 | 0.00 |
| Nutrient reservoir activity | 0 | 0.00 | 1 | 0.14 | 0 | 0.00 | 0 | 0.00 |
| Protein dimerization activity | 2 | 0.35 | 1 | 0.14 | 7 | 0.37 | 0 | 0.00 |
| Structural molecule activity | 0 | 0.00 | 0 | 0.00 | 2 | 0.11 | 20 | 11.63 |
| Transcription regulator activity | 8 | 1.41 | 4 | 0.57 | 3 | 0.16 | 3 | 1.74 |
| Transporter activity | 25 | 4.39 | 20 | 2.85 | 105 | 5.57 | 10 | 5.81 |
| Subtotal | 569 | 100.00 | 702 | 100.00 | 1886 | 100.00 | 172 | 100.00 |

**Note:** A total of 2581 genes were mapped and categorized to GO terms. Data show the number of genes that were up- or down-regulated within the categories

of biological process, cellular component and molecular function. The * represents DEGs with significant difference at a level of corrected P value <0.05.
