# Supplementary material for: Transcriptomic analysis of genes in soybean in response to Peronospora manshurica infection
Source: BMC Genomics. 2018 May 18;19:366. doi: 10.1186/s12864-018-4741-7 (PMC5960119; doi:10.1186/s12864-018-4741-7)
Supplement: Supplementary file 4 — Table S4. KEGG classification of the differentially expressed genes in HR/JL1. Note: Up- and down-regulated NCBI-Gene IDs are non-underlined and underlined, respectively. (DOCX 19 kb) [file 12864_2018_4741_MOESM4_ESM.docx]

**Table S4 KEGG classification of the differentially expressed genes in HR/JL1.**

| KEGG pathway | KEGG ID | NCBI-Gene ID |
| --- | --- | --- |
| 2-Oxocarboxylic acid metabolism | gmx01210 | 100782644 |
| alpha-Linolenic acid metabolism | gmx00592 | 100787202 |
| Amino sugar and nucleotide sugar metabolism | gmx00520 | 100780801 |
| Arginine and proline metabolism | gmx00330 | 547749 |
| Ascorbate and aldarate metabolism | gmx00053 | 100788665 |
| Biosynthesis of amino acids | gmx01230 | 732650; 100782644; 100792394 |
| Biosynthesis of secondary metabolites | gmx01110 | 100815671; 100777517; 100812820; 732650; 547749; 100782644;  100814521; 100777757; 100780801; 100815887; 100792394 |
| Carbon metabolism | gmx01200 | 100780041; 100789036; 100782793; 100792394 |
| Circadian rhythm - plant | gmx04712 | 780540; 778086 |
| Cysteine and methionine metabolism | gmx00270 | 100777207; 732650 |
| Fatty acid elongation | gmx01200 | 100792394 |
| Fatty acid metabolism | gmx01212 | 547807 |
| Fructose and mannose metabolism | gmx00051 | 100792394; 100780801 |
| Galactose metabolism | gmx00052 | 100788844; 100776791; 100815922 |
| Glycolysis / Gluconeogenesis | gmx00010 | 100780041; 100792394 |
| Glyoxylate and dicarboxylate metabolism | gmx00630 | 100789036;100782793 |
| Inositol phosphate metabolism | gmx00562 | 100788665 |
| Isoquinoline alkaloid biosynthesis | gmx00950 | 100798722 |
| Linoleic acid metabolism | gmx00591 | 100787202 |
| Metabolic pathways | gmx01100 | 100776791; 100789036; 100815671; 100777517; 100780041; 100812820; 732650; 100782793; 100803119; 547749; 100782644; 100788844; 100814521; 100780801; 547461; 100820327; 100814163; 100777757; 100794288; 100792394; 100814163; 100776789; 100794604; 100787202 |
| Nitrogen metabolism | gmx00910 | 100500448 |
| Oxidative phosphorylation | gmx00190 | 100306101 |
| Pantothenate and CoA biosynthesis | gmx00770 | 100782644 |
| Pentose phosphate pathway | gmx00030 | 100780041; 100792394 |
| Phenylalanine metabolism | gmx00360 | 100812820; 732650; 100815671 |
| Phenylalanine, tyrosine and tryptophan biosynthesis | gmx00400 | 732650 |
| Phenylpropanoid biosynthesis | gmx00940 | 100812820; 100815671 |
| Photosynthesis | gmx00195 | 100814163;100776789;100305746;100809697;100814163 |
| Photosynthesis - antenna proteins | gmx00196 | 100800374; 100820327; 100794604 |
| Plant hormone signal transduction | gmx04075 | 100792339; 100779400 |
| Plant-pathogen interaction | gmx04626 | 100810471; 100791597; 100817960 |
| Protein processing in endoplasmic reticulum | gmx04141 | 100788249; 548078 |
| Selenocompound metabolism | gmx00450 | 100777207 |
| Starch and sucrose metabolism | gmx00500 | 100803119; 100776791; 100788844 |
| Steroid biosynthesis | gmx00100 | 100814521 |
| Thiamine metabolism | gmx00730 | 100794288; 547461 |
| Tropane, piperidine and pyridine alkaloid biosynthesis | gmx00960 | 732650 |
| Tyrosine metabolism | gmx00350 | 732650 |
| Ubiquinone and other terpenoid-quinone biosynthesis | gmx00130 | 732650; 100777517 |
| Valine, leucine and isoleucine biosynthesis | gmx00290 | 100782644 |
| Valine, leucine and isoleucine degradation | gmx00280 | 100782644 |
| Zeatin biosynthesis | gmx00908 | 100777757 |

**Note：U**p- and down-regulated NCBI-Gene IDs are non-underlined and underlined, respectively.
