# Supplementary material for: Transcriptomic analysis of genes in soybean in response to Peronospora manshurica infection
Source: BMC Genomics. 2018 May 18;19:366. doi: 10.1186/s12864-018-4741-7 (PMC5960119; doi:10.1186/s12864-018-4741-7)
Supplement: Supplementary file 5 — Table S5. KEGG classification of the differentially expressed genes in the HS/KF1. Note: Up- and down-regulated NCBI-Gene IDs are non-underlined and underlined, respectively. (DOCX 19 kb) [file 12864_2018_4741_MOESM5_ESM.docx]

**Table S5 KEGG classification of the differentially expressed genes in the HS/KF1.**

| KEGG pathway | KEGG ID | NCBI-Gene ID |
| --- | --- | --- |
| alpha-Linolenic acid metabolism | gmx00592 | 100803666; 100785480 |
| Arginine and proline metabolism | gmx00330 | 100782576; 548071; 100785056 |
| beta-Alanine metabolism | gmx00410 | 100782576; 100785056; 100798722 |
| Biosynthesis of amino acids | gmx01230 | 100170686 |
| Biosynthesis of secondary metabolites | gmx01110 | 100800911 ;606705; 100808546; 547911; 100820620; 100784111; 100777336; 100789782; 100170686; 100800219; 100798722 |
| Carbon fixation in photosynthetic organisms | gmx00710 | 100792394 |
| Carotenoid biosynthesis | gmx00906 | 100779810 |
| Circadian rhythm - plant | gmx04712 | 100779346 |
| Diterpenoid biosynthesis | gmx00904 | 100806026; 100818088; 100808546 |
| Endocytosis | gmx04144 | 100777767 |
| Flavonoid biosynthesis | gmx00941 | 547911; 100800911 |
| Glycine, serine and threonine metabolism | gmx00260 | 100170686; 100798722 |
| Isoflavonoid biosynthesis | gmx00943 | 606705; 100798722 |
| Isoquinoline alkaloid biosynthesis | gmx00950 | 100798722 |
| Linoleic acid metabolism | gmx00591 | 100785480 |
| Metabolic pathways | gmx01100 | 100808931; 100527163; 100820620; 100784111; 100777336; 100789782; 548071; 100785480; 100800911; 100170686; 100803666; 100800219; 100776789; 100814163; 100798722; 100776789 |
| Peroxisome | gmx04146 | 100805057 |
| Phenylalanine metabolism | gmx00360 | 100820620; 100789782; 100800219; 100777336; 100800911; 100798722 |
| Phenylalanine, tyrosine and tryptophan biosynthesis | gmx00400 | 100170686 |
| Phenylpropanoid biosynthesis | gmx00940 | 100820620; 100789782; 100800219; 100777336; 100800911 |
| Phosphatidylinositol signaling system | gmx04070 | 548063 |
| Photosynthesis | gmx00195 | 100814163; 100776789; 100776789 |
| Photosynthesis - antenna proteins | gmx00196 | 100794944 |
| Plant hormone signal transduction | gmx04075 | 100802800;100812618;100804180;100527510;100778559;100776488;100779400;100801449;100800709;100306332; |
| Plant-pathogen interaction | gmx04626 | 100127367;100802800;100804180;547787;100817960;100306332;100527510;100778559;100812618;548063;100500561;100817960;100801449;100526987;100800709;100783572;100779760;100500636;100818929 |
| Protein processing in endoplasmic reticulum | gmx04141 | 100777767; 100780491; 100812220 |
| Purine metabolism | gmx00230 | 100527163; 100808931; 100784111; 100784833 |
| Pyrimidine metabolism | gmx00240 | 100784833 |
| Spliceosome | gmx03040 | 100777767 |
| Stilbenoid, diarylheptanoid and gingerol biosynthesis | gmx00945 | 100800911 |
| Sulfur metabolism | gmx00920 | 100799978 |
| Tropane, piperidine and pyridine alkaloid biosynthesis | gmx00960 | 100798722 |
| Tyrosine metabolism | gmx00350 | 100798722 |

**Note：U**p- and down-regulated NCBI-Gene IDs are non-underlined and underlined, respectively.
