# Supplementary material for: Transcriptomic analysis of genes in soybean in response to Peronospora manshurica infection
Source: BMC Genomics. 2018 May 18;19:366. doi: 10.1186/s12864-018-4741-7 (PMC5960119; doi:10.1186/s12864-018-4741-7)
Supplement: Supplementary file 6 — Table S6. Primers used for qRT-PCR amplification of seven DEGs. Note: F: forward primers; R: reverse primers. (DOCX 17 kb) [file 12864_2018_4741_MOESM6_ESM.docx]

**Table S6 Primers used for qRT-PCR amplification of seven DEGs.**

| Gene ID | Primers |
| --- | --- |
| Glyma08g08810  (FLS2) | F:5'-CAATTTCTTGAATGGAAGCCTC-3' |
|  | R:5'-CTGGCTAAAATCAAGAGCTCGC-3' |
| Glyma06g40710 | F:5'- TCCATATGATAATCTAGTTGG-3' |
|  | R:5'-CACCTAATGTACCTTCAAGTCG-3' |
| Glyma16g04940 | F:5'-TGTCATTGCCATCAACGACAC-3' |
|  | R:5'-CTTCAATCACCAAGTCTATCC-3' |
| Glyma04g10940 | F:5'-TATGAATCTTGAGAGTCCTGTG-3' |
|  | R:5'-TGTCTTCCAATTGAATGTTAGC-3' |
| Glyma04g20330 | F:5'-GAGAGGAGCTCTTGTGCCTAC-3' |
|  | R:5'-GCAAGCCTTACATTAGTTGC-3' |
| Glyma04G05700  (GmWRKY31) | F:5'-TGATGATGATCAATGGCCAG-3' |
|  | R:5'-TCATTGCCAACGTCTCTGC-3' |
| Glyma17g04710  (GmWRKY156) | F:5'-CTCTGAACACTACGCTGCTC-3' |
|  | R:5'-TTAAGAACTGGCTGCAGGAG-3' |

Note: F: forward primers; R: reverse primers.
